# Tau extent outperforms tau load as a predictor of neurodegeneration in Alzheimer’s disease

**DOI:** 10.1186/s13024-026-00945-1

**Published:** 2026-06-04

**Authors:** Arthur C. Macedo, Lydia Trudel, Seyyed Ali Hosseini, Joseph Therriault, Étienne Aumont, Gleb Bezgin, Nesrine Rahmouni, Jenna Stevenson, Cécile Tissot, Marcel S. Woo, Stijn Servaes, Stuart Mitchell, Jaime Fernandez-Arias, Brandon Hall, Tevy Chan, Marina Pereira Gonçalves, Pamela C. L. Ferreira, João Pedro Ferrari-Souza, Bruna Bellaver, Firoza Z. Lussier, Andréa L. Benedet, Nicholas J. Ashton, Henrik Zetterberg, Kaj Blennow, Dana L. Tudorascu, Eduardo R. Zimmer, Maxime Montembeault, Jean-Paul Soucy, Jesse Klostranec, Tharick A. Pascoal, Paolo Vitali, Pedro Rosa-Neto

**Affiliations:** 1 Translational Neuroimaging Laboratory, The McGill University Research Centre for Studies in Aging, McConnell Brain Imaging Centre (BIC), Montreal Neurological Institute, 6875 LaSalle Blvd, Montréal, QC Canada; 2 Department of Neurology and Neurosurgery, McGill University, Montréal, Québec, Canada; 3 Montreal Neurological Institute, Montréal, Québec, Canada; 4 Lawrence Berkeley National Laboratory, 1 Cyclotron Rd, Berkeley, CA USA; 5 Translational Neurodegeneration Laboratory, Department of Neurology, University Medical Center Hamburg-Eppendorf, Hamburg, Germany; 6 Department of Psychiatry, School of Medicine, University of Pittsburgh, Pittsburgh, PA USA; 7 Graduate Program in Biological Sciences: Pharmacology and Therapeutics; and Biochemistry, Universidade Federal do Rio Grande do Sul, Porto Alegre, Brazil; 8 Department of Psychiatry and Neurochemistry, Institute of Neuroscience and Physiology, the Sahlgrenska Academy, University of Gothenburg, Mölndal, Sweden; 9 Wallenberg Centre for Molecular Medicine, University of Gothenburg, Gothenburg, Sweden; 10 King’s College London, Institute of Psychiatry, Psychology and Neuroscience, Maurice Wohl Institute Clinical Neuroscience Institute, London, UK; 11 NIHR Biomedical Research Centre for Mental Health and Biomedical Research Unit for Dementia at South London and Maudsley NHS Foundation, London, UK; 12 Clinical Neurochemistry Laboratory, Sahlgrenska University Hospital, Mölndal, Sweden; 13 Department of Neurodegenerative Disease, UCL Institute of Neurology, Queen Square, London, UK; 14 UK Dementia Research Institute at UCL, London, UK; 15 Hong Kong Center for Neurodegenerative Diseases, Clear Water Bay, Hong Kong, China; 16 Wisconsin Alzheimer’s Disease Research Center, University of Wisconsin School of Medicine and Public Health, University of Wisconsin-Madison, Madison, WI USA; 17 Paris Brain Institute, ICM, Pitié-Salpêtrière Hospital, Sorbonne University, Paris, France; 18 Neurodegenerative Disorder Research Center, Division of Life Sciences and Medicine, and Department of Neurology, Institute on Aging and Brain Disorders, University of Science and Technology of China and First Affiliated Hospital of USTC, Hefei, P.R. China; 19 Brain Institute of Rio Grande do Sul, PUCRS, Porto Alegre, Brazil; 20 Department of Neurology, School of Medicine, University of Pittsburgh, Pittsburgh, PA USA; 21 The Peter O’Donnell Jr. Brain Institute (OBI), University of Texas Southwestern Medical Centre (UTSW), Dallas, TX USA

**Keywords:** Alzheimer’s disease, Neurofibrillary tangles, Positron emission tomography, Neurodegeneration

## Abstract

**Background:**

In Alzheimer’s disease (AD), tau pathology is more strongly linked to neurodegeneration than amyloid-β and better predicts brain atrophy. The spatial extent of tauopathy (SEOT) has shown promise as an earlier and more sensitive marker of AD severity than tau load, but how these complementary dimensions relate to neurodegeneration remains unclear. Here, we compared the in vivo associations of tau-PET extent versus load with cross-sectional and longitudinal neurodegeneration.

**Methods:**

We studied 367 participants across the healthy-aging to AD continuum (mean age 69.3 years; 61% female) from the TRIAD cohort who underwent [^18^F]MK-6240 tau-PET. Tau load was quantified as regional standardized uptake value ratio (SUVR), and SEOT as the proportion of abnormal voxels, within a temporal meta-region of interest (ROI) and a full-cortex ROI. Neurodegeneration markers included cortical thickness, hippocampal volume (HCV), medial temporal atrophy (MTA) visual ratings, plasma neurofilament light (NfL), and CSF total tau (t-tau). Cross-sectional associations were evaluated using multiple linear regression or covariate-adjusted Spearman correlations. We also compared local correlations of tau load and extent with cortical thickness across all cortical regions. Longitudinal predictive value for neurodegeneration was tested using linear mixed-effects models.

**Results:**

Cross-sectionally, all tau-PET metrics were significantly associated with neurodegeneration across imaging and fluid biomarkers. Full-cortex SEOT provided the best model fit for cortical thinning. SEOT outperformed tau load for associations with HCV and for predicting MTA, whereas SEOT and SUVR showed comparable associations with plasma NfL and CSF t-tau. Tau extent was equal or superior to tau load in its correlation with local cortical thickness across all cortical regions. Longitudinally, baseline full-cortex SEOT best predicted future cortical thinning, while temporal SEOT best predicted future hippocampal atrophy.

**Conclusions:**

Across cross-sectional and longitudinal analyses, tau extent provided superior predictive value for imaging-based neurodegeneration compared with tau load. By enabling a spatially unbiased, whole-brain assessment of tau burden that accommodates heterogeneous topographies, SEOT represents a promising complementary tau-PET metric for staging and tracking disease progression in AD.

**Supplementary information:**

The online version contains supplementary material available at 10.1186/s13024-026-00945-1.

## Introduction

Alzheimer’s disease (AD) is pathologically defined by the accumulation of amyloid-beta (Aβ) plaques and tau neurofibrillary tangles, which together drive neurodegeneration [1]. While Aβ is thought to contribute to neurodegenerative processes, including synaptic and neuronal loss [2], the extent of neurodegeneration correlates more strongly with tau pathology than with Aβ burden [3]. Moreover, the spatial distribution of neurodegeneration mirrors the topography of tau-PET signal more closely than that of amyloid-PET, suggesting tau’s central role in driving AD-related neuronal dysfunction associated with brain atrophy [4–7]. Longitudinal studies have further demonstrated that tau-PET metrics independently and more accurately predict neurodegeneration over time compared to amyloid-PET measures [8].

While tau plays a central role in AD progression, its accumulation can be characterized along distinct dimensions using tau-PET imaging [9], each of which may contribute differently to neurodegenerative changes. The relative contributions of these dimensions—specifically the spatial extent of tauopathy (SEOT) versus regional intensity (i.e., tau load)—to downstream neuronal loss remain to be fully elucidated. Emerging evidence suggests that SEOT metrics may represent a more reliable marker of early pathological changes than conventional tau load measures [9–11]. SEOT reflects the widespread distribution of abnormal tau-PET signal across the cortex and has demonstrated stronger associations with hippocampal atrophy and other indicators of disease severity [9–11].

This raises the question of whether SEOT could better predict neurodegeneration in AD than conventional tau load metrics, such as regional standardized uptake value ratios (SUVRs). In this study, we aimed to directly compare the associations of tau extent and load metrics with global and local neurodegeneration. Additionally, we sought to determine whether baseline tau extent serves as a better predictor of future neurodegeneration than tau load. Based on the hypothesis that the presence of tauopathy alone is sufficient to induce neurodegeneration, we posited that tau extent would outperform traditional tau load metrics in predicting tau-related neurodegeneration.

## Methods

### Study population

We examined cognitively unimpaired (CU) and cognitively impaired (CI) participants from the TRIAD cohort [12]. Participants were consecutively recruited from either the community or the McGill Centre for Studies in Aging memory clinic between November 2016 and March 2024. CU individuals were defined as those with a Clinical Dementia Rating (CDR) score of 0, indicating no objective cognitive impairment and preserved activities of daily living (ADL). CI participants included individuals diagnosed with mild cognitive impairment (MCI) or AD dementia. Similar to previous studies, we only included CI participants with biomarker evidence of AD pathology, as we aimed to investigate the healthy aging and AD continuum [13]. Individuals with MCI due to AD were characterized by a CDR score of 0.5 and relatively preserved ADL [14], while dementia participants had CDR scores of 1 to 2 and exhibited decreased performance in ADL [15]. Exclusion criteria for the TRIAD cohort included significant visual or auditory impairments that could interfere with neuropsychological assessment, inability to speak either French or English, recent traumatic brain injury or major surgery, contraindications to MRI or PET imaging, a Hachinski Ischemic Score > 4, and inadequately treated neurological, psychiatric, or systemic disorders. Detailed descriptions of the TRIAD dataset are available elsewhere [12].

The TRIAD study received ethical approval from the Montreal Neurological Institute (MNI) PET Working Committee and the Douglas Mental Health University Institute Research Ethics Board (IUSMD 16–60). All participants signed informed consent forms after being explained about all research procedures. This study was performed in accordance with the Declaration of Helsinki.

### Procedures

Participants underwent comprehensive clinical assessments for diagnostic ascertainment and imaging assessments using MRI, tau-PET, and Aβ-PET at the time point. The mean (SD) time between MRI and tau-PET was 45.3 (51.1) days. Tau-PET imaging was performed with [^18^F]MK6240, and Aβ-PET with [^18^F]AZD4694.

A subset of participants also underwent plasma neurofilament light chain (NfL) measurements with a commercially available Simoa immunoassay (Quanterix, Billerica, MA) [16] and cerebrospinal fluid (CSF) assessments of total tau (t-tau) concentration using the commercial fully automated LUMIPULSE G1200 (Fujirebio) [17]. Biomarker analyses were conducted blindly from clinical and imaging assessments at the Clinical Neurochemistry Laboratory at the Sahlgrenska University Hospital, Mölndal, Sweden.

The mean (SD) time difference between plasma collection and tau-PET imaging was 139.2 (95.7) days, while the time between lumbar punctures and tau-PET was 72.7 (66.9) days.

### Neuroimaging acquisition and processing

T1-weighted structural brain MRI at a resolution of 1-mm isotropic voxels were acquired using a 3T Siemens Magnetom scanner equipped with a standard head coil. MRI scans were processed with a custom-developed pipeline [18]. PET imaging was performed using a Siemens high-resolution research tomograph (HRRT), with a native voxel size of approximately 1.22 mm isotropic. Tau-PET with [^18^F]MK6240 and Aβ PET with [^18^F]AZD4694 were acquired during post-injection intervals of 90–110 minutes and 40–70 minutes, respectively [12]. PET image reconstruction was performed using a sequential subset expectation-maximization algorithm, generating four frames (4 × 300s for [^18^F]MK6240) or three frames (3 × 600s for [^18^F]AZD4694) per scan.

Corrections for attenuation, motion, dead time, decay, random events, and scattered coincidences were applied during reconstruction. PET images were initially aligned to each participant’s native T1-weighted MRI space using linear registration, followed by linear and nonlinear transformations to the MNI standard space. An 8-mm full-width at half maximum (FWHM) Gaussian filter was applied for spatial smoothing to improve signal-to-noise ratio and mitigate the impact of spatial uncertainty arising from PET resolution and PET–MRI registration. This choice is consistent with prior work in the same cohort indicating that smoothing of at least 6 mm is required for robust tau-PET quantification [19], particularly in small regions, and was considered appropriate given the greater sensitivity of voxel-wise analyses to noise and spatial misalignment. To mitigate potential spillover effects from off-target binding in the meninges, the meningeal regions were excluded from the PET images prior to spatial transformations and smoothing [20].

### Imaging neurodegeneration measures

Cortical thickness was quantified using FreeSurfer software version 7.4.1 [21]. Cortical thickness measurements were derived from FreeSurfer’s surface-based processing pipeline, involving multiple steps. Initially, skull stripping and intensity normalization were performed to enhance segmentation quality. Subsequently, white and gray matter structures were segmented, and the interfaces between these tissues were defined. From this segmentation, FreeSurfer generated a white matter surface (representing the boundary between white and gray matter) and a pial surface (representing the boundary between gray matter and CSF). Cortical thickness was then calculated as the distance between corresponding vertices on the white matter and pial surfaces across the cortex. Surface-area weighted average cortical thickness from all labeled cortical regions was calculated as a proxy for global neurodegeneration. We also calculated the weighted average cortical thickness of an adapted version of a previously proposed AD-signature meta-ROI composed of the entorhinal, fusiform, parahippocampal, mid-temporal, inferior-temporal, and angular gyrus [22, 23].

Additionally, T1-weighted MRI images underwent medial temporal lobe segmentation using the Automatic Segmentation of Hippocampal Subfields (ASHS) open-source software [24], following procedures described elsewhere [19]. We then derived the total hippocampal volume by summing the values for the left and right anterior and posterior hippocampi.

At baseline, a subset of participants had medial temporal lobe atrophy (MTA) scores visually assigned based on the Scheltens’ 5-point scale (from 0: no atrophy to 4: severe atrophy) [25]. For this, T1-weighted images were assessed in the coronal plane during consensus diagnostic meetings by a multidisciplinary team, which included a neuroradiologist, neurologists, geriatricians, and a neuropsychologist. We used age-based cut-offs to dichotomize the MTA scores into normal and pathological categories, following previously established thresholds [26, 27]. Specifically, scores of 0–1 were considered normal and >1 pathological for individuals under 75 years, while scores of 0–2 were considered normal and >2 pathological for individuals aged 75 years and older [26, 27]. This stratification accounts for the age-related increase in medial temporal atrophy that can occur in normal aging [26, 27].

### Tau-PET measures

Tau-PET SUVR images were derived using the inferior cerebellar gray matter as the reference region [28]. To assess tau load, we calculated average regional SUVR values. To measure SEOT (range: 0–100%), we calculated the proportion of abnormal voxels within predefined brain regions, following previously proposed methodology [9, 11]. Abnormal voxels were defined as those with values exceeding 2.5 SD above the mean of a reference group comprising 42 CU young adults (62% female) under the age of 26 years (mean age [SD]: 22.3 [1.3] years). This voxel-level thresholding method is consistent with approaches applied at the regional level in prior studies [20, 29–31] and the choice for a young reference group aligns with previous research using SEOT metrics [9]. For the main analyses, sensitivity analyses were conducted by testing alternative voxel-level thresholds (2 and 3 SD above the mean of the reference group). Following previous research [11, 32], we also evaluated an alternative reference group consisting of middle-aged and older adults defined as the 30 youngest CU Aβ− controls (60% females; mean age [SD]: 63.5 [6] years) in the study sample who had no follow-up visits. For this reference group, a threshold of 2 SD above the voxel-wise mean was applied, as previously validated against clinical and biomarker measures of AD severity across different cohorts and tau-PET tracers [11]. Mean and standard deviation voxel-wise SUVR maps of the respective reference groups are provided in Supplementary Fig. 1.

Both measures were derived from a temporal meta-ROI including the entorhinal cortex, amygdala, parahippocampal gyrus, fusiform gyrus, inferior temporal cortex, and medial temporal cortex [22]. This meta-ROI is widely used in the evaluation of in vivo tau burden [12, 33] and to establish thresholds for tau-PET positivity [34]. Additionally, we derived SEOT and average SUVR from a full-cortex ROI, as done in previous studies assessing tau extent (Supplementary Fig. 2) [35].

### Amyloid-β status assignment

Aβ status was determined using Aβ-PET global neocortical SUVR values (available for all participants), calculated with the full cerebellar gray matter as the reference region [22], or using CSF Aβ 42/40 (available for 123 participants). Aβ positivity was defined as Aβ-PET neocortical SUVR > 1.55 and/or CSF Aβ 42/40 < 0.068 [36].

### Statistical analysis

Statistical analyses were performed using R (version 4.4.1). Cross-sectional associations of tau load and SEOT with AD meta-ROI and global cortical thickness were estimated using multiple linear regression adjusted for age, sex, years of education, and neocortical Aβ-PET SUVR. All participants had complete covariate data. Linearity assumptions were checked and satisfied. Model performance was compared using the Akaike Information Criterion (AIC) [37], F-statistics, and adjusted R^2^. Differences in AIC (ΔAIC) were interpreted relative to the best-fitting model (i.e., the model with the lowest AIC): ΔAIC < 2 indicated that a model had substantial empirical support and was nearly as plausible as the best model; 4 ≤ ΔAIC < 7 indicated considerably less empirical support; and ΔAIC ≥ 10 suggested that the model was very unlikely to be the best approximation to the data [38]. To explore the independent effects of the tau burden metrics, we also tested linear models including both tau load and tau extent metrics as predictors.

For outcomes with linearity violations in the cross-sectional models (hippocampal volume, plasma NfL, and CSF t-tau), we performed Spearman’s correlations adjusted for age, sex, education, and Aβ-PET neocortical load. Intracranial volume (ICV) was additionally included as a covariate in models predicting hippocampal volume. To formally compare correlation coefficients, we used the *cocor* R package implementing Steiger’s test for comparing dependent correlations [39].

To evaluate the potential impact of ceiling effects in SEOT, we conducted sensitivity analyses for the primary cross-sectional models excluding participants who reached 100% SEOT in the temporal metric under the main SEOT definition (*n* = 5), thereby allowing greater variability in the predictor. No participants reached 100% SEOT in the cortical SEOT metric, thus no exclusions were applied for this measure.

To investigate the co-localization of tau pathology and neurodegeneration, we extracted SEOT, SUVR, and cortical thickness indices for all regions in the Desikan-Killiany-Tourville (DKT) atlas [40]. Spearman’s correlations were conducted to assess the associations between SUVR and cortical thickness and between SEOT and cortical thickness within each region. We used the *ggseg* package in R to visualize these correlation coefficients topographically. The rho coefficients from these correlations were again compared using the *cocor* R package with the Steiger’s test for dependent correlations.

To determine whether tau extent and load values increased with visual MTA scores, we performed groupwise comparisons using the Kruskal-Wallis test, followed by Dunn’s post hoc test with Bonferroni correction for multiple comparisons. We also used Spearman’s correlation to assess whether SEOT and SUVR values increased in an ordinal fashion across MTA score stages. Rho coefficients for these correlations were compared using the Steiger’s test from the *cocor* package. Moreover, we used Area Under the Curve (AUC) of Receiver Operating Characteristic (ROC) curves to evaluate the ability of temporal and cortical SUVR and SEOT to distinguish between participants classified as atrophic and non-atrophic for the MTA scale. Comparisons of AUCs were conducted using DeLong’s test.

Linear mixed-effects models were used to examine the association between baseline tau-PET measures and longitudinal trajectories of neurodegeneration imaging measures (i.e., hippocampal volume or cortical thickness), modeled as raw values across visits with follow-up time and its interaction with baseline tau-PET included as predictors. Models including random slopes for visit time were tested but resulted in singular fits, indicating negligible slope variance; therefore, final models included random intercepts only to account for correlation among repeated observations within participants. In our sample, 38% (*n* = 140) of participants had only one time point and 34% (*n* = 125) had exactly two time points; consequently, only a minority of participants had three or more observations, limiting the reliable estimation of individual slope variability. All models were adjusted for baseline age, sex, baseline education, and baseline Aβ-PET neocortical load. We also fitted models including the corresponding baseline neurodegeneration measure as a fixed-effect covariate to account for differences in disease severity at study entry. For models with hippocampal volume as the outcome, ICV was also used as a covariate. Competing models including different tau-PET metrics were compared using AIC estimated from maximum likelihood-fitted mixed-effects models. Complementary analyses to assess the impact of ceiling effects at 100% SEOT were not performed in the longitudinal sample, as only one participant with follow-up data reached 100% in the temporal meta-ROI SEOT metric, making such analyses unlikely to meaningfully influence model estimates.

To assess potential systematic dropout, we compared baseline characteristics between participants included in the longitudinal analyses and those without follow-up. Continuous variables (age, years of education, CDR, and Aβ-PET neocortical SUVR) were compared using the Wilcoxon rank-sum test, and categorical variables (sex and APOE status) using the chi-squared test.

## Results

We included 367 participants in the study (61.3% female; mean [SD] age 69.3 [8.1] years; see Table 1). The sample comprised 163 Aβ- and 58 Aβ+ CU individuals, 92 Aβ+ individuals with MCI, and 54 Aβ+ individuals with AD dementia. A total of 227 participants returned for follow-up assessments. The mean (SD) follow-up duration was 28.5 (16.3) months, ranging from 12 to 84 months. Cross-sectional plasma NfL and CSF t-tau data were available for 210 and 123 participants, respectively. Of note, five baseline and two follow-up hippocampal volume data points, as well as eight baseline global cortical thickness data points, were excluded from the initial sample of 367 participants due to failed MRI segmentation quality control.

**Table 1:** Demographic, clinical, and biomarkers characteristics of the participants

	Baseline sample (n = 367)	Sample with follow-up data (n = 227)
Age at baseline (years), mean (SD)	69.3 (8.1)	69.3 (7.7)
Female, n (%)	225 (61%)	139 (61%)
Years of education, mean (SD)	15.3 (3.6)	15.4 (3.5)
Diagnosis at baseline, n (%)	163 Aβ- CU (44%) 58 Aβ+ CU (16%) 92 Aβ+ MCI (25%) 54 Aβ+ AD dementia (15%)	114 Aβ- CU (50%) 38 Aβ+ CU (17%) 55 Aβ+ MCI (24%) 20 Aβ+ AD dementia (9%)
CDR at baseline, mean (SD)	0.3 (0.5)	0.2 (0.4)
APOE ε4 carriers*, n (%)	123 (41%)	76 (38%)
Follow-up time (months), mean (SD)	-	28.5 (16.3)

AD: Alzheimer’s disease; CU: cognitively unimpaired; MCI: mild cognitive impairment; SD: standard deviation

*65 missing values for the baseline sample and 24 missing values for sample with follow-up data

### Tau extent outperforms tau load in predicting cross-sectional imaging markers of neurodegeneration

**Fig. 1:**
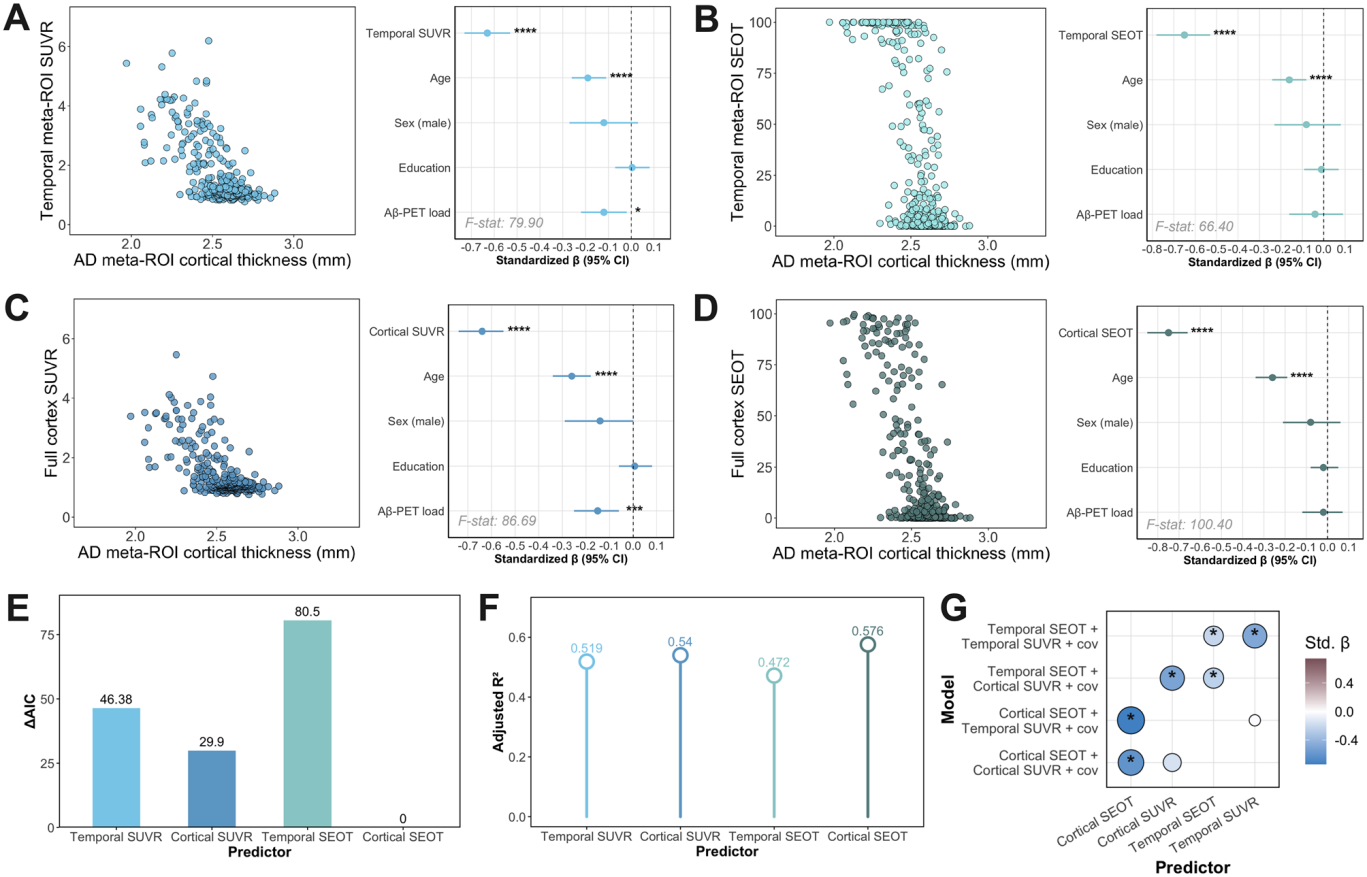
Comparative cross-sectional associations of tau extent and load with AD meta-ROI cortical thickness. Figures **A**–**D** display scatterplots and forest plots illustrating the associations between individual tau-PET metrics and AD meta-ROI cortical thickness. Linear models were adjusted for age, sex, years of education, and neocortical Aβ-PET SUVR. Figures **E** and **F** present comparisons of model fit using AIC values and adjusted R^2^, respectively, for the models shown in **A**–**D**. Figure **G** shows standardized β coefficients for each tau-PET metric when included together in a multivariable model predicting AD meta-ROI cortical thickness. *p < 0.05, **p < 0.01, ***p < 0.001,****p < 0.0001

Cross-sectionally, both temporal SUVR (Std. β = −0.63, 95% CI −0.73 to −0.53, *p* < 0.0001) and SEOT (Std. β = −0.65, 95% CI −0.78 to −0.53, *p* < 0.0001), as well as cortical SUVR (Std. β = −0.64, 95% CI −0.74 to −0.55, *p* < 0.0001) and SEOT (Std. β = −0.75, 95% CI −0.85 to −0.66, *p* < 0.0001), were significantly associated with AD meta-ROI cortical thickness, independent of age, sex, education, and Aβ-PET load (Fig.s 1A–D). Among these, cortical SEOT showed numerically higher effect size and yielded the best model fit, as indicated by higher adjusted R^2^ and lower AIC (ΔAIC > 10 relative to all other models) (Fig.s 1E–F). When cortical SEOT and SUVR metrics were included in the same model, only SEOT remained significantly associated with AD meta-ROI cortical thickness (Fig. 1G). However, when temporal SEOT was combined with either temporal SUVR or cortical SUVR in separate models, both SEOT and SUVR metrics retained statistical significance.

A similar pattern was observed for global cortical thickness: all tau-PET metrics were negatively and significantly associated with this measure (*p* < 0.0001; Supplementary Figures 3A–D). Again, the model with cortical SEOT as the main predictor showed the lowest AIC and highest adjusted R^2^ (Supplementary Figures 3E–F). In combined models, only cortical SEOT remained a significant predictor of global cortical thickness when paired with either temporal or cortical SUVR (Supplementary Figures 3 G). Meanwhile, when combining temporal SEOT with the SUVR metrics, all predictors’ associations remained significant.

**Fig. 2:**
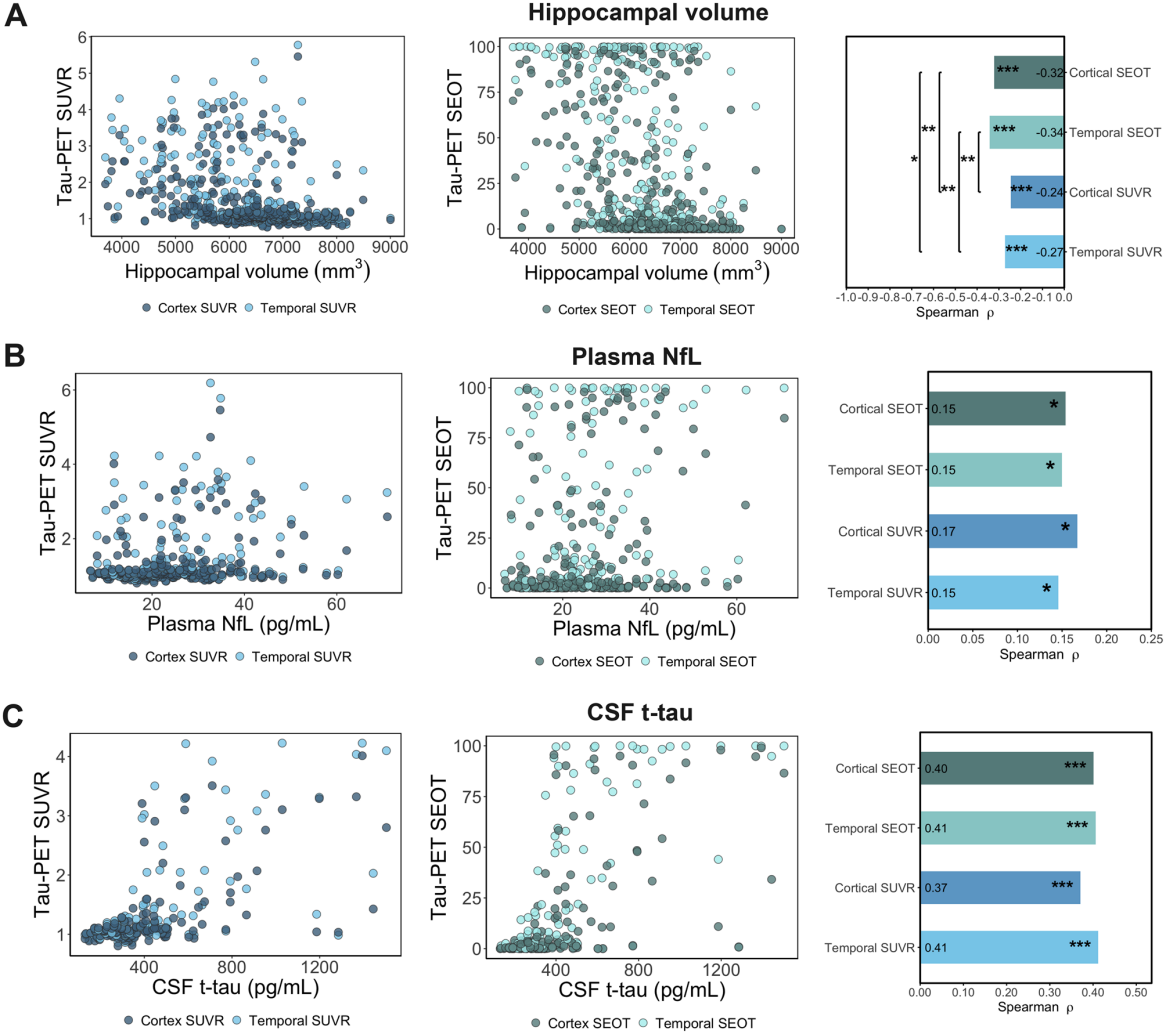
Comparative cross-sectional correlations of tau extent and load with hippocampal volume and neurodegeneration-related fluid biomarkers. Panels **A**-**C** show scatterplots (left) and bar summaries (right) of Spearman’s ρ between tau-PET metrics (SEOT and SUVR) and (**A**) hippocampal volume, (**B**) plasma neurofilament light (NfL), and (**C**) CSF total tau (t-tau). All ρ values are adjusted for age, sex, years of education, and neocortical Aβ-PET SUVR; hippocampal volume models additionally adjust for intracranial volume. Bars are annotated with ρ and significance. Pairwise comparisons of dependent correlations were performed with the Steiger’s test; significant differences are indicated where present. *p < 0.05, **p < 0.01, ***p < 0.001

Furthermore, all tau-PET metrics were significantly negatively correlated with hippocampal volume (all *p* < 0.0001). Both temporal (rho = −0.34) and cortical (rho = −0.32) SEOT exhibited stronger correlations with hippocampal volume than temporal (rho = −0.27) and cortical (rho = 0.24) SUVR (Fig. 2A).

### Tau extent and load show similar performance in predicting fluid markers of neurodegeneration

All tau-PET metrics were positively correlated with plasma NfL levels (all *p* < 0.05), with adjusted rho coefficients ranging from 0.15 to 0.17 (Fig. 2B). No significant differences were observed among the correlation coefficients across the different tau-PET measures. Similarly, temporal SUVR (rho = 0.41, *p* < 0.0001), temporal SEOT (rho = 0.41, *p* < 0.0001), cortical SUVR (rho = 0.37, *p* < 0.0001), and cortical SEOT (rho = 0.40, *p* < 0.0001) were all correlated with CSF t-tau concentrations (Fig. 2C), with no significant differences among the correlation strengths.

### Tau extent shows stronger correlations with local neurodegeneration

**Fig. 3:**
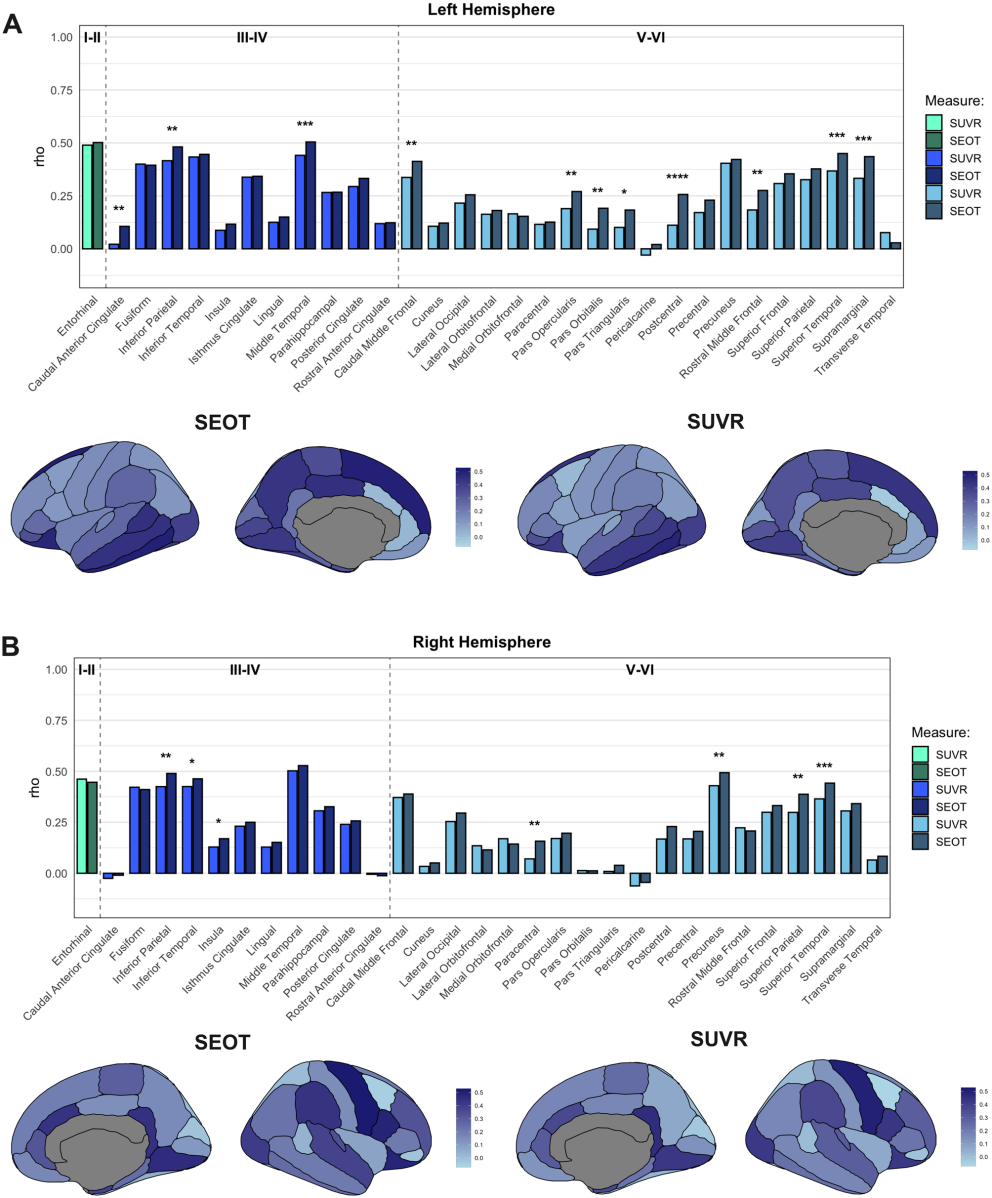
Comparison of regional correlations between tau extent, tau load, and cortical thinning. Figures **A** (left hemisphere) and **B** (right hemisphere) show the topographic distribution of Spearman correlation coefficients between cortical thickness and locally derived SEOT (tau extent) or SUVR (tau load), along with the corresponding rho values for each DKT atlas region, grouped by Braak stage. Statistical comparisons of the correlation strength between SEOT and SUVR with cortical thickness were performed using the *cocor* package; regions with significant differences are indicated. *p < 0.05, **p < 0.01, ***p < 0.001,****p < 0.0001

Tau extent was equal or superior to tau load in its correlation with regional cortical thickness across all DKT cortical regions (Fig. 3A; Supplementary Table 1). SEOT, in particular, outperformed SUVR in several intermediate to late Braak regions, with distinct hemispheric patterns observed (Fig. 3B). In the left hemisphere, SEOT showed significantly stronger correlations with cortical thinning in 11 out of 31 regions (35%), including the caudal anterior cingulate, inferior parietal, middle temporal, caudal middle frontal, pars opercularis, pars orbitalis, pars triangularis, postcentral, superior frontal, superior temporal, and supramarginal cortices. In the right hemisphere, SEOT demonstrated stronger associations in 7 regions (23%), specifically the inferior parietal, inferior temporal, insula, paracentral, precuneus, superior parietal, and superior temporal cortices.

### Tau extent shows better performance in identifying medial temporal atrophy

**Fig. 4:**
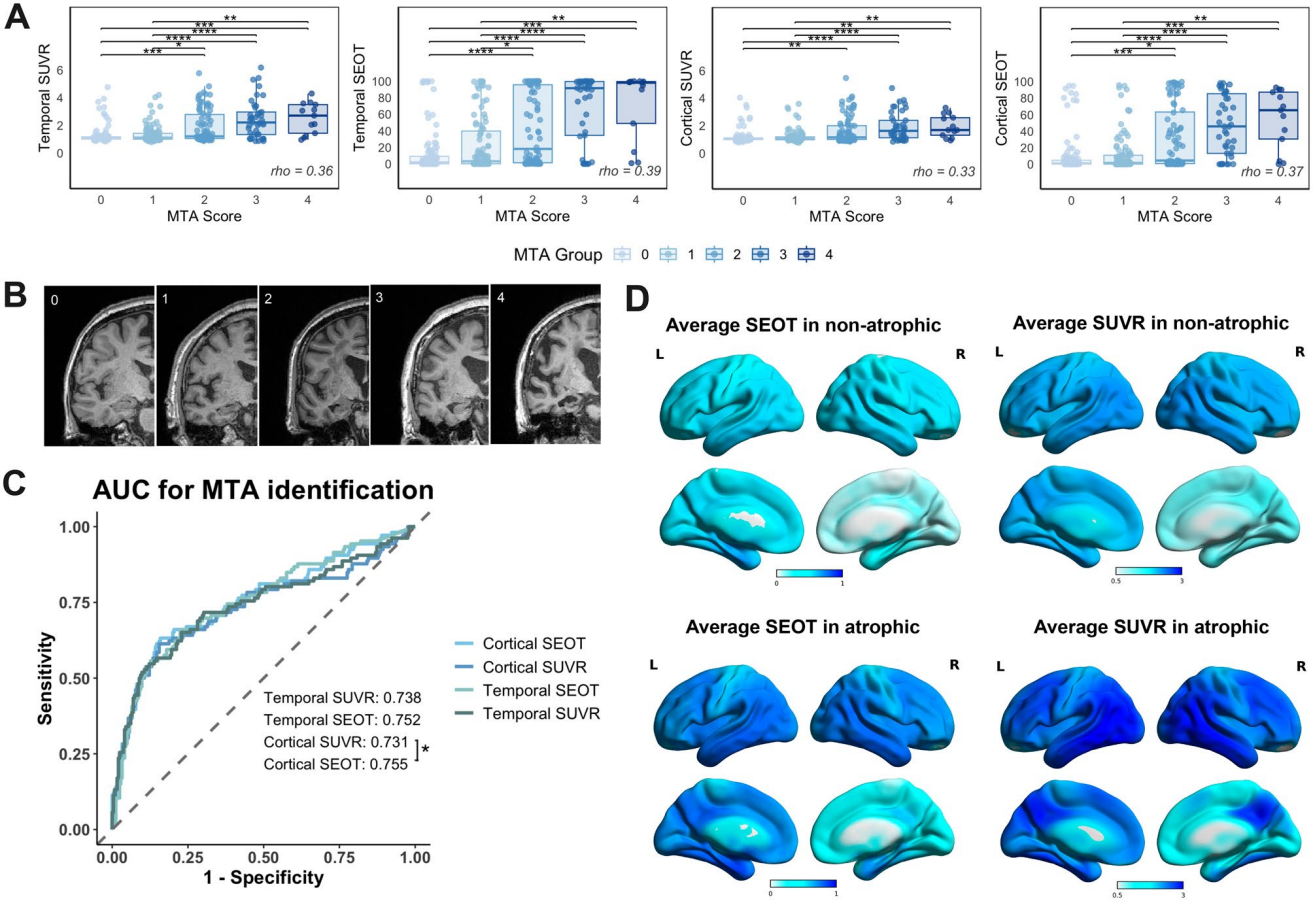
Comparative performance of tau extent and load in identifying medial temporal atrophy. Figure **A** presents boxplots of temporal and cortical SEOT and SUVR across MTA score stages. Group comparisons were performed using the Kruskal-Wallis test (all *p* < 0.0001), followed by Dunn’s post hoc test; significant pairwise differences are indicated. Figure **B** shows representative cases from the study cohort illustrating each MTA stage, providing visual examples of the progression of medial temporal atrophy. Figure **C** shows ROC curves and corresponding AUC values for each of the four tau-PET metrics in classifying individuals as atrophic based on the MTA scale. AUC comparisons were conducted using DeLong’s test, with significant differences shown. Figure **D** displays average SEOT and SUVR maps for participants stratified by MTA status (atrophic vs non-atrophic). *p < 0.05, **p < 0.01, ***p < 0.001,****p < 0.0001

MTA scores were available for 351 participants. All tau-PET metrics increased across MTA stages, with significant differences in tau levels observed only from stage 2 onward when compared to participants at stage 0, consistent with the cut-off used to define visually assessed medial temporal atrophy (Fig. 4A). Tau levels showed a stepwise increase across MTA stages, with Spearman’s rho values ranging from 0.33 to 0.39. Temporal and cortical SEOT exhibited significantly stronger correlations with MTA stage than cortical SUVR (*p* < 0.05). To illustrate this progression, representative cases for each MTA stage are shown in Fig. 4B.

To assess the ability of tau metrics to discriminate visually defined atrophy, we classified participants as atrophic (*n* = 106) or non-atrophic (*n* = 245) based on standard clinical cut-offs. SEOT measures yielded higher AUC values than their SUVR counterparts, though only the difference between cortical SEOT and cortical SUVR reached statistical significance (*p* < 0.05; Fig. 4C). For qualitative comparison, average SEOT and SUVR maps are shown in Fig. 4D. Of note, SEOT average maps can be interpreted as probabilistic representations, highlighting the likelihood of voxel-wise tau positivity within each group.

### Tau extent outperforms tau load in predicting longitudinal neurodegeneration

**Fig. 5:**
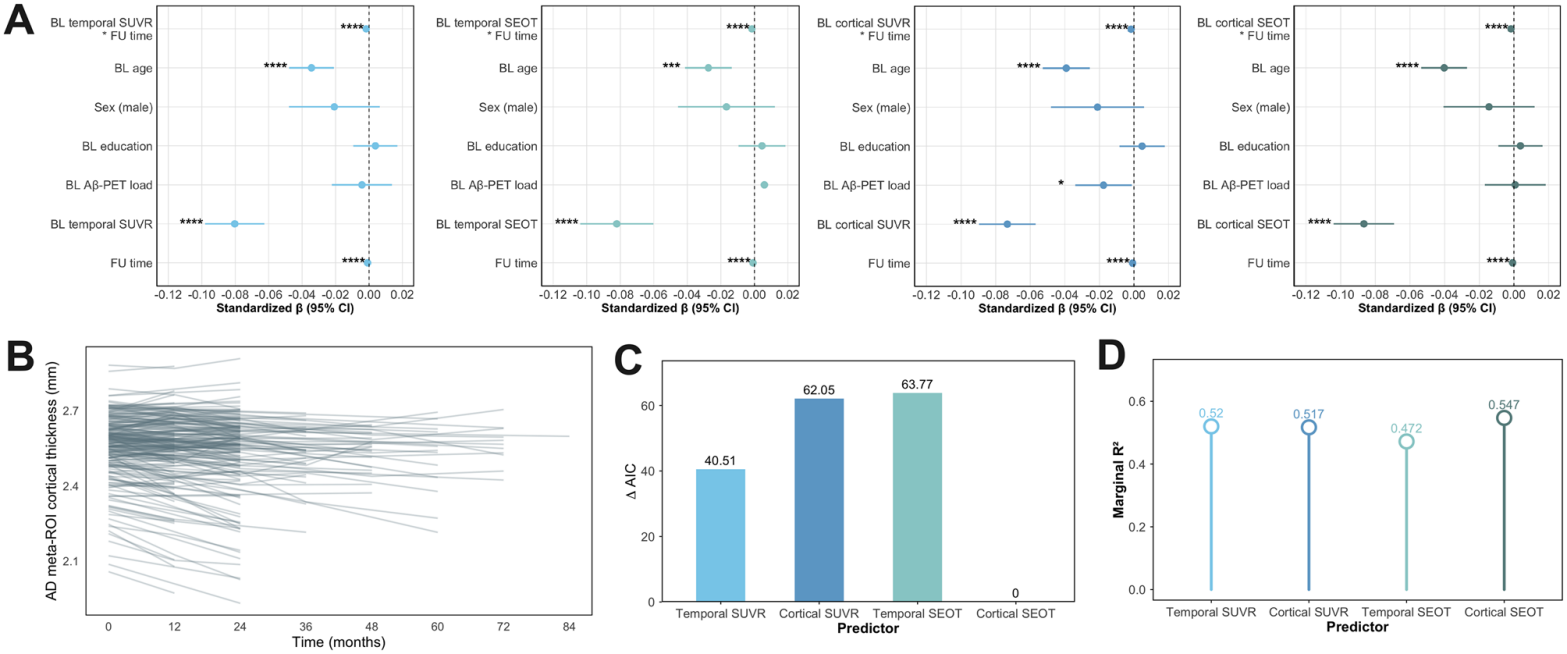
Baseline tau extent and load metrics as predictors of longitudinal AD meta-ROI cortical thickness. Figure **A** presents forest plots of effect sizes derived from linear mixed-effects models. These models evaluate the predictive value of baseline tau-PET metrics on the longitudinal cortical thinning within the AD meta-ROI. All models included an interaction term between each tau-PET metric and follow-up time, and were adjusted for baseline age, sex, baseline years of education, and baseline neocortical Aβ-PET SUVR. Figure **B** shows spaghetti plots depicting individual trajectories of AD meta-ROI cortical thickness over time. Figures **C** and **D** compare model fit using AIC values and marginal R^2^, respectively, across the different tau-PET metrics. *p < 0.05, **p < 0.01, ***p < 0.001,****p < 0.0001

We employed linear mixed-effects models to assess whether baseline tau-PET metrics could independently predict the rate of cortical and hippocampal atrophy over time. For AD-specific longitudinal cortical thinning, interaction terms between all tau-PET metrics and follow-up time exhibited comparable effect sizes, each reaching statistical significance (Fig. 5A–D). Notably, the cortical SEOT model demonstrated a substantially better fit, reflected by consistently lower AIC values (ΔAIC > 10 relative to other models), indicating superior predictive performance.

When global cortical thickness was the outcome, the interaction effects of cortical SUVR (standardized β = −0.00086, 95% CI: −0.001 to −0.0007, *p* < 0.0001) and cortical SEOT (standardized β = −0.00088, 95% CI: −0.001 to −0.0007, *p* < 0.0001) with time of follow-up were numerically higher than those of their temporal region counterparts (Supplementary Figure 4). Correspondingly, models incorporating cortical SUVR and SEOT showed improved AIC values, with the cortical SEOT model achieving the best fit.

**Fig. 6:**
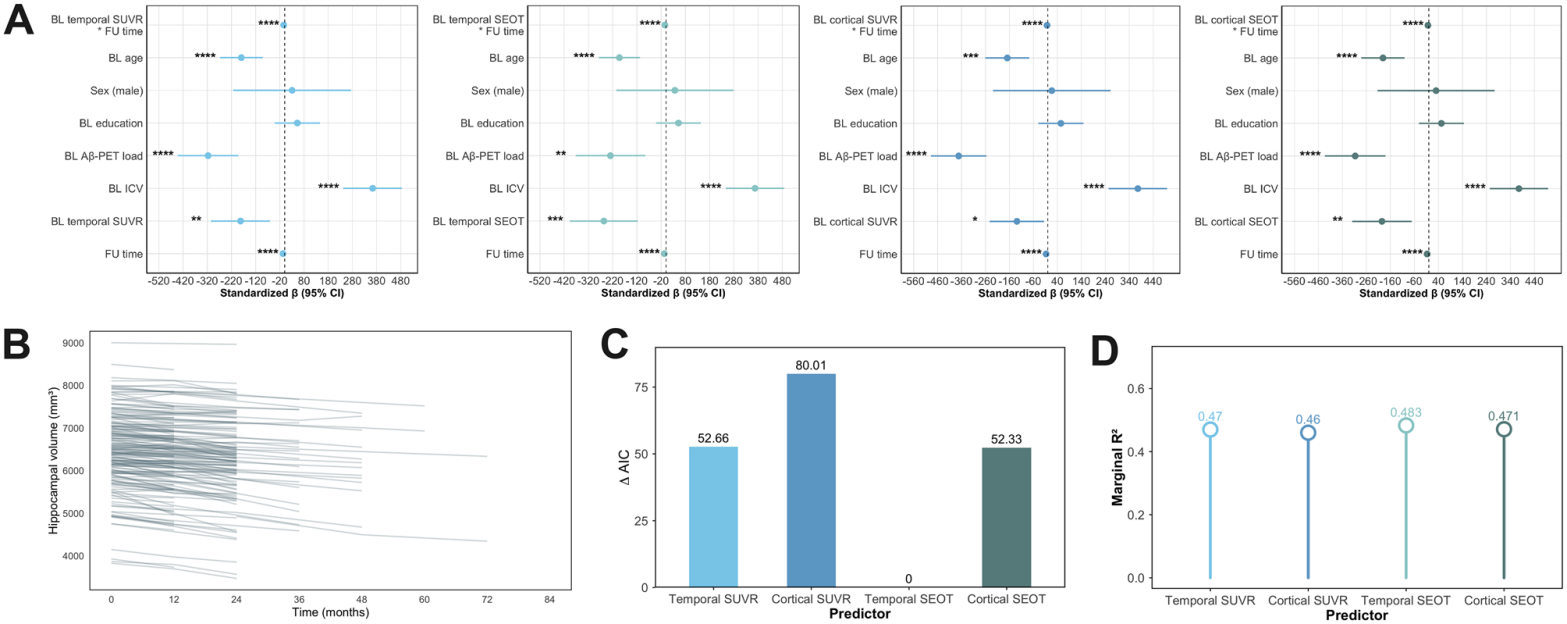
Baseline tau extent and load metrics as predictors of longitudinal hippocampal volume. Figure **A** presents forest plots of effect sizes derived from linear mixed-effects models. These models evaluate the predictive value of baseline tau-PET metrics on the longitudinal hippocampal atrophy. All models included an interaction term between each tau-PET metric and follow-up time, and were adjusted for baseline age, sex, baseline years of education, baseline neocortical Aβ-PET SUVR, and intracranial volume. Figure **B** shows spaghetti plots depicting individual trajectories of hippocampal volume over time. Figures **C** and **D** compare model fit using AIC values and marginal R^2^, respectively, across the different tau-PET metrics. *p < 0.05, **p < 0.01, ***p < 0.001,****p < 0.0001

Consistent with cross-sectional findings, the temporal SEOT model provided the best fit for predicting longitudinal hippocampal atrophy, exhibiting the largest effect size for its interaction with follow-up time (standardized β = −4.6, 95% CI: −5.3 to −3.8, *p* < 0.0001; Fig. 6). This model also outperformed others by a substantial margin, with an AIC at least 52.33 points lower than alternative models.

Including the baseline value of the corresponding neurodegeneration measure as a fixed-effect covariate did not materially change the results, with all baseline tau-PET predictors remaining significant predictors of future neurodegeneration (Supplementary Figures 5–7). Cortical SEOT continued to show the best model fit for predicting cortical thinning, while temporal SEOT provided the best fit for predicting hippocampal atrophy.

### Sensitivity analyses

In sensitivity analyses excluding participants with temporal SEOT equal to 100%, derived at the 2.5 SD threshold using young controls as the reference, findings remained consistent. Linear model parameters and comparative results showed no meaningful changes when using either AD meta-ROI (Supplementary Figure 8A–C) or global cortical thickness (Supplementary Figure 9A–C) as outcomes. Similarly, only minimal differences were observed in correlation coefficients between tau-PET metrics and hippocampal volume, plasma NfL, and CSF t-tau, with no changes in pairwise comparisons across metrics (Supplementary Figure 10A–C).

Moreover, sensitivity analyses using alternative SEOT calculation approaches demonstrated significant cross-sectional associations for all SEOT variants in linear models with AD meta-ROI and global cortical thickness as outcomes (all *p* < 0.0001; Supplementary Figure 11A-B). In AIC comparisons, the four cortical SEOT variants yielded the lowest AIC values among the ten tau-PET predictors evaluated, all with ΔAIC > 10 relative to the remaining models (Supplementary Figure 11C-D). Among cortical SEOT variants, AIC values were largely comparable across definitions, except for the 2-SD metric derived using the younger reference group, which showed poorer fit.

SEOT metrics derived using different methodological approaches were also significantly associated with hippocampal volume, plasma NfL, and CSF t-tau (Supplementary Figures 12A-C). In statistical comparisons of Spearman’s ρ coefficients, SEOT metrics, particularly temporal SEOT variants, generally showed stronger correlations with hippocampal volume than SUVR measures (Supplementary Table 2). SEOT metrics derived using the older reference group tended to show weaker correlations with hippocampal volume than those derived using younger controls, although these differences rarely remained significant after FDR correction. For fluid biomarkers, the only nominal differences observed were that temporal SEOT (2 SD) showed a stronger correlation with CSF t-tau than temporal SEOT calculated using the older reference group, and cortical SEOT (3 SD) showed a stronger correlation than cortical SEOT (2.5 SD); however, these differences did not survive correction for multiple comparisons (Supplementary Table 3). No significant differences were observed for plasma NfL (Supplementary Table 4).

When testing whether baseline tau-PET predicted longitudinal neurodegeneration, sensitivity analyses showed that SEOT metrics derived using alternative methodological approaches were significant predictors of future AD meta-ROI cortical thinning, global cortical thinning, and hippocampal atrophy (Supplementary Figure 13A–C). In models predicting AD meta-ROI cortical thickness, cortical SEOT calculated using thresholds of 2 and 2.5 SD above the young reference group yielded the lowest AIC values among all tau-PET predictors (ΔAIC > 10 relative to competing models; Supplementary Figure 13D). Notably, models including all SEOT predictors except temporal SEOT (2 SD) showed stronger empirical support than those including SUVR metrics. When global cortical thickness was used as the outcome, models including cortical SEOT metrics consistently outperformed those including SUVR or temporal SEOT metrics (ΔAIC > 10 relative to competing models; Supplementary Figure 13E). Finally, when hippocampal volume was used as the outcome, temporal SEOT (2 SD) yielded the lowest AIC value (ΔAIC > 10 relative to all other predictors), although all temporal SEOT metrics showed clear advantages over models including other tau-PET metrics (Supplementary Figure 13F).

Taken together, these findings indicate that the main results were robust to variations in SEOT definition, including the choice of threshold and reference group, with consistent effect directions and largely preserved relative performance across metrics. While differences in model fit were observed across SEOT parameterizations, these did not alter the overall pattern of associations. Notably, the optimal SEOT parameterization showed some outcome-specific variation, with certain threshold–reference group combinations providing better model fit for hippocampal atrophy versus cortical thinning, suggesting that SEOT may be flexibly tuned to capture distinct aspects of neurodegeneration.

### Baseline differences according to follow-up status

By comparing baseline characteristics of participants with (*n* = 227) and without (*n* = 100) follow-up visits, we observed no differences in age, sex, years of education, or APOE status (Supplementary Table 5). However, participants without follow-up exhibited higher baseline CDR scores (*p* < 0.001) and Aβ-PET neocortical SUVR (*p* = 0.002).

## Discussion

In this study, we compared tau extent and load, measured using a second-generation tau-PET tracer with subnanomolar affinity, as predictors of current and future neurodegeneration in the aging and AD spectrum. Overall, tau extent metrics demonstrated superior performance in predicting neurodegeneration as measured by imaging visual and quantitative assessments. In contrast, tau extent and load showed comparable associations with plasma NfL and CSF t-tau concentrations. These findings build on prior evidence underscoring the clinical relevance of tau and amyloid extent metrics [9–11, 32, 35, 41–43] and suggest that extent-based measures may offer unique value as prognostic tools. Furthermore, their robust associations with multiple markers of disease severity support their utility for advancing our understanding of the pathophysiological mechanisms underlying AD.

Our findings align with prior research indicating that tau pathology is a reliable and independent predictor of neurodegeneration in AD [8, 44]. Notably, we observed a stronger regional association between tau extent and cortical thinning, which may indicate a more temporally proximal relationship between tau accumulation and neuronal damage than is typically inferred from conventional SUVR-based measures. While previous models have linked neurodegeneration to high SUVR values reflecting tau accumulation intensity, our findings suggest that the spatial extent of tau-PET signal above a pathological threshold may more effectively capture the initial presence of pathological tau capable of triggering neurodegenerative changes, aligning with prior reports that highlight the sensitivity of this metric to early-stage pathology [9–11]. This interpretation is consistent with mechanistic evidence showing that hyperphosphorylated tau not only loses its normal role in stabilizing neuronal microtubules but also acts as a pathological seed, recruiting soluble tau into aggregates [45–49]. In this context, SEOT may more accurately reflect the early presence of pathological tau capable of initiating these seeding events, whereas regional SUVR may better index the cumulative burden of tau pathology resulting from aggregation. Indeed, tau seeding activity has been shown to precede overt tau deposition in humans [50–52], potentially serving not only as a driver of further tau accumulation but also as a contributor to early, localized neurodegeneration.

Our study compared tau extent and load metrics derived from two ROIs: the widely used temporal meta-ROI, traditionally applied for quantifying and diagnosing tau burden in AD [22], and a non-regionally biased full cortex ROI. While the temporal meta-ROI demonstrates good diagnostic performance in differentiating clinical groups [12] and in identifying AD-related pathology at autopsy [53], it is inherently limited by its anatomical scope, encompassing only regions corresponding to Braak stages I through IV [54, 55]. Although this regional focus aligns with established neuropathological criteria for AD diagnosis [56], it does not capture the full spectrum of tau distribution patterns observed in the disease [57]. Indeed, increasing evidence from both neuropathological and imaging studies has identified the presence of Braak non-conformant cases (i.e., individuals whose tau pathology deviates from the canonical staging), highlighting the clinical and biological heterogeneity of AD [55, 58, 59]. Accurately staging and diagnosing these atypical cases remains a significant challenge. Unlike post-mortem pathology, tau-PET imaging provides the opportunity for in vivo whole-brain assessment, circumventing the spatial limitations of histological sampling. Limiting tau burden quantification to predefined ROIs may not only underutilize the comprehensive nature of tau-PET but may also reduce sensitivity to detect atypical or spatially diffuse tau patterns that contribute to neurodegeneration [60, 61]. In this context, SEOT emerges as a promising metric for global assessment of cortical tau burden. This approach could help prevent underestimation of focal yet clinically relevant tau-PET signals that might otherwise be diluted in regional averaging, especially across large ROIs such as the whole cortex [62].

In our study, SEOT outperformed regional SUVR in the prediction of global and AD-related cortical thinning, as well as hippocampal atrophy. Moreover, SEOT metrics achieved the highest performance for distinguishing between atrophic and non-atrophic individuals based on the MTA scale [25], a visual rating tool widely used in clinical practice, highlighting the translational potential of these findings. By contrast, no significant differences were observed among tau-PET metrics in their associations with fluid biomarkers. This may reflect the systemic and non-regional nature of plasma NfL, which is influenced by multiple sources of axonal injury and thus less sensitive to subtle differences between tau extent and load measures [63, 64]. For CSF t-tau, the lack of differences likely reflects its role as a global non-specific marker of tau species rather than neurodegeneration per se [64, 65], as evidenced by its recent removal from the “(N)” category in the revised NIA-AA research framework [63]. Taken together with recent studies showing stronger associations of SEOT with other AD severity markers [9–11] and its improved accuracy in identifying early disease stages [10, 11], these results support SEOT as a robust metric with potential value in both clinical and research applications.

A key strength of our study is the use of a highly sensitive second-generation tau-PET tracer, combined with multiple complementary neurodegeneration measures, to validate our findings within a well-characterized and ongoing longitudinal cohort. However, several limitations should be acknowledged. First, the study sample predominantly comprised White individuals recruited in Canada, which may limit the generalizability of the results to more diverse populations. Second, due to a limited number of participants with more than two follow-up visits, our linear mixed-effects models did not include random slopes, which restricts the ability to fully capture individual variability in the trajectory of cortical atrophy. Replication in other datasets with extended follow-up durations is encouraged to better model individual neurodegenerative progression. Additionally, the SEOT metric was derived using arbitrary thresholds, a known limitation of threshold-based methods that may impact the sensitivity and specificity of detection. To minimize this bias, we adopted cutoffs previously validated at both the ROI and voxel levels [11, 20, 29–31] and conducted sensitivity analyses to assess how different cutoffs and reference groups affected the main results. Beyond threshold selection, another methodological consideration relates to the level of spatial smoothing applied. While the use of an 8 mm kernel improves signal-to-noise ratio and mitigates voxel-level misregistration, it may also influence the spatial specificity of voxel-wise extent estimates. Future studies should systematically evaluate the impact of different smoothing levels on SEOT quantification. It is also important to note that while SEOT provides a valuable measure of tau burden by capturing the proportion of abnormal voxels, this proportion can reach a ceiling of 100%, beyond which further changes in tau burden are not detectable. This saturation effect could limit the utility of SEOT in predicting neurodegeneration in more advanced disease stages or in populations with widespread tau pathology. By employing a full cortex ROI and including a sample ranging from CU individuals to those with moderate dementia, we minimized this concern, as few participants approached, but none reached, the 100% cortical SEOT saturation threshold. This issue is more likely to arise when using smaller ROIs, particularly regions affected earlier in the disease process, such as the temporal meta-ROI, for which we observed some cases reaching 100%. However, this does not preclude its use, as demonstrated by our results, and ROI choice may be guided by the clinical severity of the study population. Moreover, a SEOT value of 100% remains informative, as it reflects complete regional involvement rather than merely a limitation of the metric. Furthermore, although CI participants without evidence of amyloid pathology were excluded, co-pathologies associated with other causes of neurodegeneration are common in AD [66] and should be considered in future studies when such data are available. Finally, as reported in other longitudinal AD studies [67], participants without follow-up exhibited a higher baseline disease burden, which may bias longitudinal estimates by underrepresenting individuals with more advanced pathology. However, in this ongoing cohort without systematically recorded attrition reasons, the absence of follow-up likely reflects a combination of true dropout and participants who had not yet reached their scheduled follow-up visit rather than selective attrition.

In conclusion, our findings support the notion that the spatial extent of tau pathology is more strongly associated with local neurodegenerative changes in AD than the local load of tau deposition. Moreover, tau extent metrics demonstrate reliable predictive value for both current and future neurodegeneration, offering the possibility for a regionally agnostic approach to assessing tau burden. This has important implications not only for advancing our imaging-based understanding of AD pathophysiology but also for enhancing our ability to evaluate individuals with atypical patterns of tau accumulation.

## Electronic supplementary material

Below is the link to the electronic supplementary material.

Supplementary Material 1

## Data Availability

All requests for raw and analysed data and materials will be promptly reviewed by McGill University to verify if the request is subject to any intellectual property or confidentiality obligations. Anonymized data will be shared upon request to the study’s senior author from a qualified academic investigator for the sole purpose of replicating the procedures and results presented in this article. Any data and materials that can be shared will be released via a material transfer agreement. Data are not publicly available due to information that could compromise the privacy of research participants. Related documents, including study protocol and informed consent forms, can similarly be made available upon request.
