# Supplementary material for: Tau extent outperforms tau load as a predictor of neurodegeneration in Alzheimer’s disease
Source: Mol Neurodegener. 2026 Jun 4;21:45. doi: 10.1186/s13024-026-00945-1 (PMC13463738; doi:10.1186/s13024-026-00945-1)
Supplement: Supplementary file 1 [file 13024_2026_945_MOESM1_ESM.docx]

**Supplementary Table 1.** Regional comparison of Spearman correlations between tau-PET SUVR and SEOT with local cortical thinning across cortical brain regions.

| **Hemisphere** | **Region** | **SUVR-thickness rho** | **p** | **SEOT-thickness rho** | **p** | **Rho comparison p** |
| --- | --- | --- | --- | --- | --- | --- |
| left | caudal anterior cingulate | 0.02 | 0.68 | 0.11 | 0.04 | 0.004 |
| left | caudal middle frontal | 0.34 | < 0.0001 | 0.41 | < 0.0001 | 0.006 |
| left | cuneus | 0.11 | 0.04 | 0.12 | 0.02 | 0.61 |
| left | entorhinal | 0.49 | < 0.0001 | 0.50 | < 0.0001 | 0.37 |
| left | fusiform | 0.40 | < 0.0001 | 0.39 | < 0.0001 | 0.75 |
| left | inferior parietal | 0.42 | < 0.0001 | 0.48 | < 0.0001 | 0.005 |
| left | inferior temporal | 0.43 | < 0.0001 | 0.45 | < 0.0001 | 0.48 |
| left | insula | 0.09 | 0.09 | 0.12 | 0.03 | 0.10 |
| left | isthmus cingulate | 0.34 | < 0.0001 | 0.34 | < 0.0001 | 0.87 |
| left | lateral occipital | 0.22 | < 0.0001 | 0.26 | < 0.0001 | 0.11 |
| left | lateral orbitofrontal | 0.16 | 0.002 | 0.18 | 0.0005 | 0.43 |
| left | lingual | 0.13 | 0.02 | 0.15 | 0.004 | 0.33 |
| left | medial orbitofrontal | 0.17 | 0.001 | 0.15 | 0.003 | 0.65 |
| left | middle temporal | 0.44 | < 0.0001 | 0.50 | < 0.0001 | 0.0009 |
| left | paracentral | 0.12 | 0.03 | 0.13 | 0.02 | 0.75 |
| left | parahippocampal | 0.27 | < 0.0001 | 0.27 | < 0.0001 | 0.93 |
| left | pars opercularis | 0.19 | 0.0003 | 0.27 | < 0.0001 | 0.005 |
| left | pars orbitalis | 0.09 | 0.08 | 0.19 | 0.0002 | 0.003 |
| left | pars triangularis | 0.10 | 0.05 | 0.18 | 0.0004 | 0.01 |
| left | pericalcarine | -0.03 | 0.57 | 0.02 | 0.70 | 0.14 |
| left | postcentral | 0.11 | 0.03 | 0.26 | < 0.0001 | < 0.0001 |
| left | posterior cingulate | 0.29 | < 0.0001 | 0.33 | < 0.0001 | 0.12 |
| left | precentral | 0.17 | 0.00099 | 0.23 | < 0.0001 | 0.06 |
| left | precuneus | 0.40 | < 0.0001 | 0.42 | < 0.0001 | 0.44 |
| left | rostral anterior cingulate | 0.12 | 0.02 | 0.12 | 0.02 | 0.86 |
| left | rostral middlefrontal | 0.18 | 0.0004 | 0.28 | < 0.0001 | 0.003 |
| left | superior frontal | 0.31 | < 0.0001 | 0.35 | < 0.0001 | 0.09 |
| left | superior parietal | 0.33 | < 0.0001 | 0.38 | < 0.0001 | 0.06 |
| left | superior temporal | 0.37 | < 0.0001 | 0.45 | < 0.0001 | 0.0002 |
| left | supramarginal | 0.33 | < 0.0001 | 0.44 | < 0.0001 | 0.0003 |
| left | transverse temporal | 0.08 | 0.14 | 0.03 | 0.59 | 0.17 |
| right | caudal anterior cingulate | -0.02 | 0.63 | -0.01 | 0.85 | 0.56 |
| right | caudal middle frontal | 0.37 | < 0.0001 | 0.39 | < 0.0001 | 0.52 |
| right | cuneus | 0.03 | 0.53 | 0.05 | 0.33 | 0.58 |
| right | entorhinal | 0.46 | < 0.0001 | 0.45 | < 0.0001 | 0.26 |
| right | fusiform | 0.42 | < 0.0001 | 0.41 | < 0.0001 | 0.51 |
| right | inferior parietal | 0.42 | < 0.0001 | 0.49 | < 0.0001 | 0.003 |
| right | inferior temporal | 0.43 | < 0.0001 | 0.46 | < 0.0001 | 0.04 |
| right | insula | 0.13 | 0.01 | 0.17 | 0.001 | 0.04 |
| right | isthmus cingulate | 0.23 | < 0.0001 | 0.25 | < 0.0001 | 0.42 |
| right | lateral occipital | 0.25 | < 0.0001 | 0.30 | < 0.0001 | 0.16 |
| right | lateral orbitofrontal | 0.13 | 0.0097 | 0.11 | 0.03 | 0.39 |
| right | lingual | 0.13 | 0.01 | 0.15 | 0.004 | 0.35 |
| right | medial orbitofrontal | 0.17 | 0.001 | 0.14 | 0.006 | 0.37 |
| right | middle temporal | 0.50 | < 0.0001 | 0.53 | < 0.0001 | 0.17 |
| right | paracentral | 0.07 | 0.18 | 0.16 | 0.002 | 0.009 |
| right | parahippocampal | 0.31 | < 0.0001 | 0.33 | < 0.0001 | 0.15 |
| right | pars opercularis | 0.17 | 0.001 | 0.20 | 0.0002 | 0.36 |
| right | pars orbitalis | 0.01 | 0.80 | 0.01 | 0.82 | 0.97 |
| right | pars triangularis | 0.01 | 0.86 | 0.04 | 0.46 | 0.37 |
| right | pericalcarine | -0.06 | 0.24 | -0.04 | 0.39 | 0.59 |
| right | postcentral | 0.17 | 0.001 | 0.23 | < 0.0001 | 0.07 |
| right | posterior cingulate | 0.24 | < 0.0001 | 0.26 | < 0.0001 | 0.48 |
| right | precentral | 0.17 | 0.001 | 0.21 | < 0.0001 | 0.22 |
| right | precuneus | 0.43 | < 0.0001 | 0.49 | < 0.0001 | 0.005 |
| right | rostral anterior cingulate | -0.01 | 0.92 | -0.01 | 0.82 | 0.79 |
| right | rostral middlefrontal | 0.22 | < 0.0001 | 0.21 | < 0.0001 | 0.60 |
| right | superior frontal | 0.30 | < 0.0001 | 0.33 | < 0.0001 | 0.18 |
| right | superior parietal | 0.30 | < 0.0001 | 0.39 | < 0.0001 | 0.002 |
| right | superior temporal | 0.36 | < 0.0001 | 0.44 | < 0.0001 | 0.0004 |
| right | supramarginal | 0.31 | < 0.0001 | 0.34 | < 0.0001 | 0.15 |
| right | transverse temporal | 0.07 | 0.21 | 0.08 | 0.11 | 0.59 |

Statistical comparisons of rho coefficients were performed using Steiger’s test for dependent correlations, implemented in the *cocor* R package.

**Supplementary Table 2.** Statistical comparison of Spearman’s correlation coefficients between hippocampal volume and tau-PET SUVR and SEOT metrics derived using different thresholds and reference groups

| Measure 1 | Measure 2 | rho 1 | rho 2 | Nominal p | FDR p |
| --- | --- | --- | --- | --- | --- |
| Temporal SEOT (3 SD) | Cortical SUVR | -0.35 | -0.24 | 0.001 | 0.02 |
| Temporal SEOT (2.5 SD) | Cortical SUVR | -0.34 | -0.24 | 0.002 | 0.02 |
| Temporal SEOT (3 SD) | Temporal SUVR | -0.35 | -0.27 | 0.002 | 0.02 |
| Temporal SEOT (2 SD) | Cortical SUVR | -0.33 | -0.24 | 0.002 | 0.02 |
| Temporal SEOT (2.5 SD) | Temporal SUVR | -0.34 | -0.27 | 0.003 | 0.02 |
| Temporal SEOT (2 SD) | Temporal SUVR | -0.33 | -0.27 | 0.003 | 0.02 |
| Cortical SEOT (2 SD) | Cortical SUVR | -0.31 | -0.24 | 0.004 | 0.02 |
| Cortical SEOT (2.5 SD) | Cortical SUVR | -0.32 | -0.24 | 0.004 | 0.02 |
| Cortical SEOT (older ref) | Cortical SEOT (2.5 SD) | -0.27 | -0.32 | 0.007 | 0.03 |
| Temporal SEOT (3 SD) | Cortical SEOT (older ref) | -0.35 | -0.27 | 0.007 | 0.03 |
| Cortical SEOT (older ref) | Temporal SEOT (2.5 SD) | -0.27 | -0.34 | 0.01 | 0.06 |
| Cortical SEOT (3 SD) | Cortical SUVR | -0.31 | -0.24 | 0.02 | 0.06 |
| Temporal SEOT (older ref) | Cortical SUVR | -0.32 | -0.24 | 0.02 | 0.06 |
| Cortical SEOT (2 SD) | Cortical SEOT (older ref) | -0.31 | -0.27 | 0.02 | 0.06 |
| Temporal SEOT (2 SD) | Cortical SEOT (older ref) | -0.33 | -0.27 | 0.02 | 0.07 |
| Cortical SEOT (3 SD) | Cortical SEOT (older ref) | -0.31 | -0.27 | 0.04 | 0.11 |
| Cortical SEOT (2.5 SD) | Temporal SUVR | -0.32 | -0.27 | 0.05 | 0.13 |
| Cortical SEOT (3 SD) | Temporal SEOT (3 SD) | -0.31 | -0.35 | 0.06 | 0.16 |
| Temporal SEOT (older ref) | Temporal SUVR | -0.32 | -0.27 | 0.07 | 0.16 |
| Cortical SEOT (2 SD) | Temporal SEOT (3 SD) | -0.31 | -0.35 | 0.08 | 0.17 |
| Cortical SEOT (2 SD) | Temporal SUVR | -0.31 | -0.27 | 0.08 | 0.17 |
| Cortical SEOT (older ref) | Temporal SEOT (older ref) | -0.27 | -0.32 | 0.09 | 0.18 |
| Temporal SEOT (3 SD) | Temporal SEOT (older ref) | -0.35 | -0.32 | 0.09 | 0.18 |
| Temporal SEOT (2 SD) | Temporal SEOT (3 SD) | -0.33 | -0.35 | 0.11 | 0.20 |
| Temporal SEOT (2 SD) | Temporal SEOT (2.5 SD) | -0.33 | -0.34 | 0.11 | 0.20 |
| Cortical SEOT (2 SD) | Temporal SEOT (2.5 SD) | -0.31 | -0.34 | 0.12 | 0.21 |
| Cortical SEOT (3 SD) | Temporal SUVR | -0.31 | -0.27 | 0.14 | 0.23 |
| Cortical SEOT (2 SD) | Cortical SEOT (2.5 SD) | -0.31 | -0.32 | 0.14 | 0.23 |
| Cortical SEOT (3 SD) | Temporal SEOT (2.5 SD) | -0.31 | -0.34 | 0.15 | 0.23 |
| Temporal SEOT (3 SD) | Cortical SEOT (2.5 SD) | -0.35 | -0.32 | 0.15 | 0.23 |
| Cortical SUVR | Temporal SUVR | -0.24 | -0.27 | 0.17 | 0.24 |
| Cortical SEOT (older ref) | Cortical SUVR | -0.27 | -0.24 | 0.19 | 0.27 |
| Temporal SEOT (older ref) | Temporal SEOT (2.5 SD) | -0.32 | -0.34 | 0.21 | 0.28 |
| Cortical SEOT (2 SD) | Temporal SEOT (2 SD) | -0.31 | -0.33 | 0.23 | 0.31 |
| Cortical SEOT (3 SD) | Cortical SEOT (2.5 SD) | -0.31 | -0.32 | 0.26 | 0.33 |
| Cortical SEOT (2.5 SD) | Temporal SEOT (2.5 SD) | -0.32 | -0.34 | 0.26 | 0.33 |
| Temporal SEOT (3 SD) | Temporal SEOT (2.5 SD) | -0.35 | -0.34 | 0.30 | 0.36 |
| Temporal SEOT (2 SD) | Cortical SEOT (3 SD) | -0.33 | -0.31 | 0.34 | 0.40 |
| Temporal SEOT (2 SD) | Temporal SEOT (older ref) | -0.33 | -0.32 | 0.48 | 0.55 |
| Temporal SEOT (2 SD) | Cortical SEOT (2.5 SD) | -0.33 | -0.32 | 0.55 | 0.62 |
| Cortical SEOT (2 SD) | Temporal SEOT (older ref) | -0.31 | -0.32 | 0.80 | 0.88 |
| Cortical SEOT (3 SD) | Temporal SEOT (older ref) | -0.31 | -0.32 | 0.82 | 0.88 |
| Temporal SEOT (older ref) | Cortical SEOT (2.5 SD) | -0.32 | -0.32 | 0.86 | 0.90 |
| Cortical SEOT (older ref) | Temporal SUVR | -0.27 | -0.27 | 0.93 | 0.94 |
| Cortical SEOT (2 SD) | Cortical SEOT (3 SD) | -0.31 | -0.31 | 0.94 | 0.94 |

Statistical comparisons were performed using Steiger’s test for dependent correlations, implemented in the *cocor* R package. Older ref: reference group composed of middle-aged and older adults; SD: standard deviation.

**Supplementary Table 3.** Statistical comparison of Spearman’s correlation coefficients between plasma NfL and tau-PET SUVR and SEOT metrics derived using different thresholds and reference groups

| Measure 1 | Measure 2 | rho 1 | rho 2 | Nominal p | FDR p |
| --- | --- | --- | --- | --- | --- |
| Cortical SEOT (3 SD) | Cortical SEOT (2.5 SD) | 0.17 | 0.15 | 0.29 | 0.99 |
| Temporal SEOT (2 SD) | Cortical SEOT (3 SD) | 0.14 | 0.17 | 0.43 | 0.99 |
| Cortical SEOT (2 SD) | Cortical SEOT (3 SD) | 0.15 | 0.17 | 0.44 | 0.99 |
| Cortical SUVR | Temporal SUVR | 0.17 | 0.15 | 0.47 | 0.99 |
| Cortical SEOT (3 SD) | Temporal SEOT (2.5 SD) | 0.17 | 0.15 | 0.55 | 0.99 |
| Temporal SEOT (2 SD) | Cortical SUVR | 0.14 | 0.17 | 0.56 | 0.99 |
| Cortical SEOT (3 SD) | Temporal SUVR | 0.17 | 0.15 | 0.57 | 0.99 |
| Temporal SEOT (2 SD) | Cortical SEOT (older ref) | 0.14 | 0.16 | 0.59 | 0.99 |
| Cortical SEOT (3 SD) | Temporal SEOT (3 SD) | 0.17 | 0.15 | 0.60 | 0.99 |
| Temporal SEOT (2 SD) | Temporal SEOT (2.5 SD) | 0.14 | 0.15 | 0.62 | 0.99 |
| Cortical SEOT (older ref) | Temporal SUVR | 0.16 | 0.15 | 0.62 | 0.99 |
| Cortical SEOT (2 SD) | Cortical SEOT (older ref) | 0.15 | 0.16 | 0.63 | 0.99 |
| Cortical SEOT (2 SD) | Cortical SUVR | 0.15 | 0.17 | 0.64 | 0.99 |
| Temporal SEOT (2 SD) | Temporal SEOT (3 SD) | 0.14 | 0.15 | 0.66 | 0.99 |
| Temporal SEOT (2 SD) | Cortical SEOT (2.5 SD) | 0.14 | 0.15 | 0.68 | 0.99 |
| Temporal SEOT (2 SD) | Temporal SEOT (older ref) | 0.14 | 0.16 | 0.69 | 0.99 |
| Temporal SEOT (2.5 SD) | Cortical SUVR | 0.15 | 0.17 | 0.69 | 0.99 |
| Cortical SEOT (older ref) | Cortical SEOT (2.5 SD) | 0.16 | 0.15 | 0.72 | 0.99 |
| Cortical SEOT (older ref) | Temporal SEOT (2.5 SD) | 0.16 | 0.15 | 0.72 | 0.99 |
| Cortical SEOT (2.5 SD) | Cortical SUVR | 0.15 | 0.17 | 0.73 | 0.99 |
| Cortical SEOT (2 SD) | Temporal SEOT (2 SD) | 0.15 | 0.14 | 0.74 | 0.99 |
| Cortical SEOT (3 SD) | Temporal SEOT (older ref) | 0.17 | 0.16 | 0.74 | 0.99 |
| Temporal SEOT (3 SD) | Cortical SUVR | 0.15 | 0.17 | 0.75 | 0.99 |
| Temporal SEOT (older ref) | Temporal SUVR | 0.16 | 0.15 | 0.76 | 0.99 |
| Temporal SEOT (3 SD) | Cortical SEOT (older ref) | 0.15 | 0.16 | 0.78 | 0.99 |
| Cortical SEOT (2.5 SD) | Temporal SUVR | 0.15 | 0.15 | 0.81 | 0.99 |
| Temporal SEOT (older ref) | Cortical SUVR | 0.16 | 0.17 | 0.82 | 0.99 |
| Temporal SEOT (older ref) | Temporal SEOT (2.5 SD) | 0.16 | 0.15 | 0.82 | 0.99 |
| Cortical SEOT (2 SD) | Temporal SUVR | 0.15 | 0.15 | 0.84 | 0.99 |
| Temporal SEOT (3 SD) | Temporal SEOT (2.5 SD) | 0.15 | 0.15 | 0.85 | 0.99 |
| Temporal SEOT (3 SD) | Temporal SUVR | 0.15 | 0.15 | 0.85 | 0.99 |
| Cortical SEOT (older ref) | Temporal SEOT (older ref) | 0.16 | 0.16 | 0.85 | 0.99 |
| Cortical SEOT (2.5 SD) | Temporal SEOT (2.5 SD) | 0.15 | 0.15 | 0.86 | 0.99 |
| Cortical SEOT (2 SD) | Cortical SEOT (2.5 SD) | 0.15 | 0.15 | 0.86 | 0.99 |
| Cortical SEOT (3 SD) | Cortical SEOT (older ref) | 0.17 | 0.16 | 0.87 | 0.99 |
| Temporal SEOT (3 SD) | Temporal SEOT (older ref) | 0.15 | 0.16 | 0.89 | 0.99 |
| Temporal SEOT (2.5 SD) | Temporal SUVR | 0.15 | 0.15 | 0.90 | 0.99 |
| Cortical SEOT (2 SD) | Temporal SEOT (older ref) | 0.15 | 0.16 | 0.91 | 0.99 |
| Cortical SEOT (older ref) | Cortical SUVR | 0.16 | 0.17 | 0.91 | 0.99 |
| Cortical SEOT (2 SD) | Temporal SEOT (2.5 SD) | 0.15 | 0.15 | 0.92 | 0.99 |
| Temporal SEOT (3 SD) | Cortical SEOT (2.5 SD) | 0.15 | 0.15 | 0.94 | 0.99 |
| Temporal SEOT (older ref) | Cortical SEOT (2.5 SD) | 0.16 | 0.15 | 0.95 | 0.99 |
| Temporal SEOT (2 SD) | Temporal SUVR | 0.14 | 0.15 | 0.96 | 0.99 |
| Cortical SEOT (3 SD) | Cortical SUVR | 0.17 | 0.17 | 0.98 | 0.99 |
| Cortical SEOT (2 SD) | Temporal SEOT (3 SD) | 0.15 | 0.15 | 0.99 | 0.99 |

Statistical comparisons were performed using Steiger’s test for dependent correlations, implemented in the *cocor* R package. NfL: neurofilament light chain; older ref: reference group composed of middle-aged and older adults; SD: standard deviation.

**Supplementary Table 4.** Statistical comparison of Spearman’s correlation coefficients between CSF t-tau and tau-PET SUVR and SEOT metrics derived using different thresholds and reference groups

| Measure 1 | Measure 2 | rho 1 | rho 2 | Nominal p | FDR p |
| --- | --- | --- | --- | --- | --- |
| Temporal SEOT (2 SD) | Temporal SEOT (older ref) | 0.42 | 0.36 | 0.04 | 0.92 |
| Cortical SEOT (3 SD) | Cortical SEOT (2.5 SD) | 0.37 | 0.40 | 0.049 | 0.92 |
| Temporal SEOT (2 SD) | Temporal SEOT (2.5 SD) | 0.42 | 0.41 | 0.11 | 0.92 |
| Temporal SEOT (2 SD) | Cortical SEOT (3 SD) | 0.42 | 0.37 | 0.13 | 0.92 |
| Temporal SEOT (older ref) | Temporal SEOT (2.5 SD) | 0.36 | 0.41 | 0.16 | 0.92 |
| Temporal SEOT (older ref) | Temporal SUVR | 0.36 | 0.41 | 0.17 | 0.92 |
| Cortical SEOT (2 SD) | Cortical SEOT (3 SD) | 0.40 | 0.37 | 0.18 | 0.92 |
| Temporal SEOT (3 SD) | Temporal SEOT (older ref) | 0.40 | 0.36 | 0.19 | 0.92 |
| Temporal SEOT (2 SD) | Cortical SUVR | 0.42 | 0.37 | 0.25 | 0.92 |
| Temporal SEOT (older ref) | Cortical SEOT (2.5 SD) | 0.36 | 0.40 | 0.27 | 0.92 |
| Cortical SEOT (2 SD) | Temporal SEOT (older ref) | 0.40 | 0.36 | 0.27 | 0.92 |
| Cortical SUVR | Temporal SUVR | 0.37 | 0.41 | 0.27 | 0.92 |
| Cortical SEOT (older ref) | Temporal SEOT (older ref) | 0.40 | 0.36 | 0.28 | 0.92 |
| Temporal SEOT (2 SD) | Temporal SEOT (3 SD) | 0.42 | 0.40 | 0.32 | 0.92 |
| Cortical SEOT (3 SD) | Temporal SEOT (2.5 SD) | 0.37 | 0.41 | 0.33 | 0.92 |
| Cortical SEOT (3 SD) | Cortical SEOT (older ref) | 0.37 | 0.40 | 0.34 | 0.92 |
| Cortical SEOT (3 SD) | Temporal SEOT (3 SD) | 0.37 | 0.40 | 0.37 | 0.92 |
| Cortical SEOT (2 SD) | Cortical SUVR | 0.40 | 0.37 | 0.38 | 0.92 |
| Cortical SEOT (older ref) | Cortical SUVR | 0.40 | 0.37 | 0.39 | 0.92 |
| Cortical SEOT (3 SD) | Temporal SUVR | 0.37 | 0.41 | 0.41 | 0.92 |
| Temporal SEOT (2 SD) | Cortical SEOT (2.5 SD) | 0.42 | 0.40 | 0.43 | 0.92 |
| Cortical SEOT (2 SD) | Temporal SEOT (2 SD) | 0.40 | 0.42 | 0.48 | 0.94 |
| Cortical SEOT (2.5 SD) | Cortical SUVR | 0.40 | 0.37 | 0.48 | 0.94 |
| Temporal SEOT (2.5 SD) | Cortical SUVR | 0.41 | 0.37 | 0.51 | 0.96 |
| Temporal SEOT (3 SD) | Cortical SUVR | 0.40 | 0.37 | 0.57 | 0.99 |
| Temporal SEOT (2 SD) | Cortical SEOT (older ref) | 0.42 | 0.40 | 0.58 | 0.99 |
| Temporal SEOT (2 SD) | Temporal SUVR | 0.42 | 0.41 | 0.71 | 0.99 |
| Cortical SEOT (3 SD) | Temporal SEOT (older ref) | 0.37 | 0.36 | 0.71 | 0.99 |
| Temporal SEOT (older ref) | Cortical SUVR | 0.36 | 0.37 | 0.78 | 0.99 |
| Cortical SEOT (2.5 SD) | Temporal SUVR | 0.40 | 0.41 | 0.81 | 0.99 |
| Cortical SEOT (older ref) | Temporal SUVR | 0.40 | 0.41 | 0.82 | 0.99 |
| Cortical SEOT (2 SD) | Temporal SUVR | 0.40 | 0.41 | 0.84 | 0.99 |
| Cortical SEOT (2 SD) | Cortical SEOT (2.5 SD) | 0.40 | 0.40 | 0.85 | 0.99 |
| Temporal SEOT (3 SD) | Temporal SUVR | 0.40 | 0.41 | 0.85 | 0.99 |
| Temporal SEOT (3 SD) | Temporal SEOT (2.5 SD) | 0.40 | 0.41 | 0.87 | 0.99 |
| Cortical SEOT (2.5 SD) | Temporal SEOT (2.5 SD) | 0.40 | 0.41 | 0.89 | 0.99 |
| Temporal SEOT (2.5 SD) | Temporal SUVR | 0.41 | 0.41 | 0.89 | 0.99 |
| Cortical SEOT (older ref) | Temporal SEOT (2.5 SD) | 0.40 | 0.41 | 0.94 | 0.99 |
| Cortical SEOT (2 SD) | Temporal SEOT (2.5 SD) | 0.40 | 0.41 | 0.95 | 0.99 |
| Cortical SEOT (2 SD) | Cortical SEOT (older ref) | 0.40 | 0.40 | 0.96 | 0.99 |
| Temporal SEOT (3 SD) | Cortical SEOT (2.5 SD) | 0.40 | 0.40 | 0.96 | 0.99 |
| Cortical SEOT (older ref) | Cortical SEOT (2.5 SD) | 0.40 | 0.40 | 0.97 | 0.99 |
| Cortical SEOT (2 SD) | Temporal SEOT (3 SD) | 0.40 | 0.40 | 0.98 | 0.99 |
| Cortical SEOT (3 SD) | Cortical SUVR | 0.37 | 0.37 | 0.99 | 0.99 |
| Temporal SEOT (3 SD) | Cortical SEOT (older ref) | 0.40 | 0.40 | 0.99 | 0.99 |

Statistical comparisons were performed using Steiger’s test for dependent correlations, implemented in the *cocor* R package. CSF: cerebrospinal fluid; older ref: reference group composed of middle-aged and older adults; SD: standard deviation; t-tau: total tau.

**Supplementary Table 5. Comparison of baseline demographic, clinical, and biomarkers characteristics of the participants with and without follow-up data.**

|  | **Sample without follow-up (n=140)** | **Sample with follow-up (n=227)** | **p-value** |
| --- | --- | --- | --- |
| **Age (years), mean (SD)** | 69.1 (8.7) | 69.3 (7.6) | 0.87 |
| **Female, n (%)** | 86 (61%) | 139 (61%) | 1.00 |
| **Years of education, mean (SD)** | 15.1 (3.9) | 15.4 (3.5) | 0.27 |
| **CDR, mean (SD)** | 0.4 (0.6) | 0.2 (0.4) | <0.001 |
| **APOE ε4 carriers*, n (%)** | 47 (47%) | 76 (38%) | 0.15 |
| **Aβ-PET neocortical SUVR, mean (SD)** | 2.00 (0.7) | 1.7 (0.5) | 0.002 |

* 40 missing values in the no follow-up group and 25 in the follow-up group.

**Supplementary Figure 1.** **Mean and standard deviation voxelwise SUVR maps of the reference groups used to derive SEOT.** The figure shows brain maps generated from cognitively unimpaired Aβ− individuals in the young reference group (**n = 42**) and in the middle- to older-aged reference group (**n = 30**).

**Supplementary Figure 2. Regions of Interest Used to Derive Tau Extent and Load Metrics.** This figure provides a visual representation of the anatomical regions included in the tau-PET analyses. The temporal meta-ROI comprises the entorhinal cortex, amygdala, parahippocampal gyrus, fusiform gyrus, inferior temporal cortex, and medial temporal cortex. The full cortex ROI includes all cortical regions.

**Supplementary Figure 3. Comparative cross-sectional associations of tau extent and load with global cortical thickness.** Figures A–D display scatterplots and forest plots illustrating the associations between individual tau-PET metrics and global average cortical thickness. Linear models were adjusted for age, sex, years of education, and neocortical Aβ-PET SUVR. Figures E and F present comparisons of model fit using AIC values and adjusted R², respectively, for the models shown in A–D. Figure G shows standardized β coefficients for each tau-PET metric when included together in a multivariable model predicting global average cortical thickness. *p<.05, **p<.01, ***p<.001,****p<.0001

**Supplementary Figure 4. Baseline tau extent and load metrics as predictors of longitudinal global cortical thickness.** Figure A presents forest plots of effect sizes derived from linear mixed-effects models. These models evaluate the predictive value of baseline tau-PET metrics on the longitudinal cortical thinning within the whole cortex. All models included an interaction term between each tau-PET metric and follow-up time, and were adjusted for baseline age, sex, baseline years of education, and baseline neocortical Aβ-PET SUVR. Figure B shows spaghetti plots depicting individual trajectories of global cortical thickness over time. Figures C and D compare model fit using AIC values and marginal R², respectively, across the different tau-PET metrics. *p<.05, **p<.01, ***p<.001,****p<.0001

**Supplementary Figure 5. Baseline tau extent and load metrics as predictors of longitudinal AD meta-ROI cortical thickness, in models additionally adjusted for baseline neurodegeneration.** Figure A presents forest plots of effect sizes derived from linear mixed-effects models. These models evaluate the predictive value of baseline tau-PET metrics on the longitudinal cortical thinning within the AD meta-ROI. All models included an interaction term between each tau-PET metric and follow-up time, and were adjusted for baseline age, sex, baseline years of education, baseline neocortical Aβ-PET SUVR, and baseline AD meta-ROI cortical thickness. Figures B and C compare model fit using AIC values and marginal R², respectively, across the different tau-PET metrics. *p<.05, **p<.01, ***p<.001,****p<.0001

**Supplementary Figure 6. Baseline tau extent and load metrics as predictors of longitudinal global cortical thickness, in models additionally adjusted for baseline neurodegeneration.** Figure A presents forest plots of effect sizes derived from linear mixed-effects models. These models evaluate the predictive value of baseline tau-PET metrics on the longitudinal cortical thinning within the whole cortex. All models included an interaction term between each tau-PET metric and follow-up time, and were adjusted for baseline age, sex, baseline years of education, baseline neocortical Aβ-PET SUVR, and baseline global cortical thickness. Figures B and C compare model fit using AIC values and marginal R², respectively, across the different tau-PET metrics. *p<.05, **p<.01, ***p<.001,****p<.0001

**Supplementary Figure 7. Baseline tau extent and load metrics as predictors of longitudinal hippocampal volume, in models additionally adjusted for baseline neurodegeneration.** Figure A presents forest plots of effect sizes derived from linear mixed-effects models. These models evaluate the predictive value of baseline tau-PET metrics on the longitudinal hippocampal atrophy. All models included an interaction term between each tau-PET metric and follow-up time, and were adjusted for baseline age, sex, baseline years of education, baseline neocortical Aβ-PET SUVR, baseline hippocampal volume, and intracranial volume. Figures B and C compare model fit using AIC values and marginal R², respectively, across the different tau-PET metrics. *p<.05, **p<.01, ***p<.001,****p<.0001

**Supplementary Figure 8. Comparative cross-sectional associations of tau extent and load with AD meta-ROI cortical thickness, excluding individuals with temporal SEOT equal to 100%.** Figure A displays forest plots illustrating the associations between individual tau-PET metrics and AD meta-ROI cortical thickness. Linear models were adjusted for age, sex, years of education, and neocortical Aβ-PET SUVR. Figures B and C present comparisons of model fit using AIC values and adjusted R², respectively, for the models shown in A. *p<.05, **p<.01, ***p<.001,****p<.0001

**Supplementary Figure 9. Comparative cross-sectional associations of tau extent and load with global cortical thickness, excluding individuals with temporal SEOT equal to 100%.** Figure A displays forest plots illustrating the associations between individual tau-PET metrics and AD meta-ROI cortical thickness. Linear models were adjusted for age, sex, years of education, and neocortical Aβ-PET SUVR. Figures B and C present comparisons of model fit using AIC values and adjusted R², respectively, for the models shown in A. *p<.05, **p<.01, ***p<.001,****p<.0001

**Supplementary Figure 10. Comparative cross-sectional correlations of tau extent and load with hippocampal volume and neurodegeneration-related fluid biomarkers, excluding individuals with temporal SEOT equal to 100%.** Panels A-C showbar summaries of Spearman’s ρ between tau-PET metrics (SEOT and SUVR) and (A) hippocampal volume, (B) plasma neurofilament light (NfL), and (C) CSF total tau (t-tau). All ρ values are adjusted for age, sex, years of education, and neocortical Aβ-PET SUVR; hippocampal volume models additionally adjust for intracranial volume. Bars are annotated with ρ and significance. Pairwise comparisons of dependent correlations were performed with the Steiger’s test; significant differences are indicated where present. *p<.05, **p<.01, ***p<.001

**Supplementary Figure 11.** **Comparative cross-sectional associations of tau load and tau extent, derived using different methodologies, with AD meta-ROI and global cortical thickness.** Figures A and B show forest plots of effect sizes for the associations between individual tau-PET metrics (as main predictors in separate linear models) and AD meta-ROI cortical thickness (A) and global cortical thickness (B). SEOT metrics were derived using a young control group at different thresholds (2, 2.5, and 3 SD above the reference mean), as well as using a middle- to older-aged reference group (“older ref”) at a threshold of 2 SD. Models were adjusted for age, sex, years of education, and neocortical Aβ-PET SUVR. Figures C and D show model fit comparisons for the models presented in A and B, respectively, based on AIC. ****p < 0.0001.

**Supplementary Figure 12.** **Comparative cross-sectional correlations of tau load and tau extent, derived using different methodologies, with hippocampal volume and neurodegeneration-related fluid biomarkers.** Panels A-C showbar summaries of Spearman’s ρ between tau-PET metrics (SEOT and SUVR) and (A) hippocampal volume, (B) plasma neurofilament light (NfL), and (C) CSF total tau (t-tau). SEOT metrics were derived using a young control group at different thresholds (2, 2.5, and 3 SD above the reference mean), as well as using a middle- to older-aged reference group (“older ref”) at a threshold of 2 SD. All ρ values are adjusted for age, sex, years of education, and neocortical Aβ-PET SUVR; hippocampal volume models additionally adjust for intracranial volume. Bars are annotated with ρ and significance. ****p<.0001

**Supplementary Figure 13.** **Comparison of baseline tau load and tau extent, derived using different methodologies, as predictors of longitudinal neurodegeneration.** Figures A–C show forest plots of effect sizes for the interaction between each tau-PET metric and follow-up time in linear mixed-effects models fitted separately to predict AD meta-ROI cortical thickness (A), global cortical thickness (B), and hippocampal volume (C). SEOT metrics were derived using a young control group at thresholds of 2, 2.5, and 3 SD above the reference mean, as well as using a middle- to older-aged reference group (“older ref”) at a threshold of 2 SD. All models were adjusted for baseline age, sex, baseline years of education, and baseline neocortical Aβ-PET SUVR. Models for hippocampal volume (C) were additionally adjusted for intracranial volume. Figures D–F show model fit comparisons, based on AIC, corresponding to the models in A–C, respectively. ****p < 0.0001.
